# Dopamine depletion in Parkinson’s increases directed but not random exploration

**DOI:** 10.1126/sciadv.aee6556

**Published:** 2026-08-05

**Authors:** Björn Meder, Martha Sterf, Charley M. Wu, Matthias Guggenmos

**Affiliations:** ^1^ Institute for Mind, Brain, and Behavior, Health and Medical University, Olympischer Weg 1, 14471 Potsdam, Germany.; ^2^ Medical School Berlin, Rüdesheimer Str. 50, 14197 Berlin, Germany.; ^3^ Human and Machine Cognition Lab, University of Tübingen, Maria-von-Linden-Str. 6, 72074 Tübingen, Germany.; ^4^ Institute of Psychology, Technical University of Darmstadt, Karolinenplatz 5, 64289 Darmstadt, Germany.; ^5^ Hessian.AI, Landwehrstraße 50A, 64293 Darmstadt, Germany.; ^6^ Bernstein Center for Computational Neuroscience Berlin, Philippstraße 13, 10115 Berlin, Germany.

## Abstract

We investigated how patients with Parkinson’s disease (PD) manage the explore-exploit trade-off in a structured reward-learning task. Patients were tested either on (*n* = 34) or off (*n* = 34) dopaminergic medication (levodopa), with age-matched polyneuropathy patients serving as controls (*n* = 35). Behaviorally, patients off medication showed marked learning and decision-making deficits, characterized by overexploration (excessive sampling of novel options) and insufficient exploitation (re-selection of previously rewarding options). To clarify the underlying mechanisms, we applied a Gaussian process–upper confidence bound (GP-UCB) model that combines generalization across spatially similar options with uncertainty-directed exploration (targeted sampling of uncertain options) and random exploration (undirected choice variability). Modeling results showed that patients off medication exhibited increased uncertainty-directed exploration compared to both controls and patients with PD on medication, whereas random exploration did not differ across groups. Relative to controls, patients off medication also showed reduced generalization. In contrast, directed exploration and generalization in patients on medication were comparable to the control group. Our findings highlight how dopamine depletion in PD affects reward learning under uncertainty, suggesting a key role of dopamine in exploration and generalization.

## Teaser

Levodopa restores the balance between exploration and exploitation in Parkinson’s disease.

## INTRODUCTION

Parkinson’s disease (PD) involves degeneration of the dopaminergic system, which is central not only for motor control but also for learning, decision-making, and exploratory behavior (*1*–*6*). The dopamine deficit in PD impairs several aspects of cognitive function, including the ability to learn from feedback and adapt to changes in reward contingencies (*7*–*9*). While simpler associative learning mechanisms can be preserved, patients show marked deficits in tasks that require building and using an internal model of the environment (*10*). Dopaminergic medication in the form of levodopa can alleviate these impairments to some degree, especially when learning from reward feedback (*7*, *11*–*13*).

Critically, PD also interferes with the ability to navigate the exploration-exploitation trade-off (*14*–*16*), which requires balancing exploring promising novel options and exploiting known high-reward options. Whether deciding between ordering your favorite dish or trying something new, or choosing a new travel destination over a familiar favorite, the fundamental challenge is to choose between what is known to work and discovering something even better (*17*–*19*). Investigating how PD influences the exploration-exploitation trade-off is therefore important from both a clinical and theoretical perspective, as it links dopaminergic dysfunction to central mechanisms of learning and decision-making.

An essential paradigm for investigating explore-exploit problems are bandit tasks, in which participants learn about choice options with uncertain outcomes through trial and error (*20*–*23*). In each trial, learners must choose between the available options, balancing the exploration of new options with the exploitation of known options. While many tasks in the literature have independent options where the outcome of one decision does not provide information about other options (*24*–*28*), natural environments are often characterized by structural regularities (*29*–*31*). For instance, spatially proximate areas often yield similar foraging outcomes (*32*), and if two dishes share similar ingredients, trying one helps predict the taste of the other (*33*). Such latent regularities can be used to generalize beyond direct experiences and adapt exploratory decisions to environmental structure (*34*).

Exploratory behavior can be decomposed into two distinct mechanisms: random exploration, in which choices vary due to undirected decision noise, and uncertainty-directed exploration, in which uncertain options are sampled because of their information value (*29*, *35*–*37*). As adaptive decision-making under uncertainty requires balancing both forms of exploration, a central question is how exactly the dopaminergic system shapes exploratory behavior. For instance, one previous study found that levodopa selectively reduced uncertainty-directed exploration in healthy participants, while random exploration remained largely unaffected (*26*). Moreover, the influence of valence on information acquisition can be reduced under levodopa (*38*). These findings suggest that dopamine modulates the valuation processes that govern exploratory decisions. However, the precise role of dopamine in exploratory behavior is still poorly understood.

### Goals and scope

It is well established that patients with PD exhibit pronounced deficits in reinforcement learning, typically attributed to dopaminergic degeneration (*7*–*13*). To better understand how dopamine and medication specifically shape exploratory behavior in PD, we used a behavioral paradigm that captures two critical features of real-life exploration: the abundance of choice options and the presence of hidden structure that can be leveraged to search efficiently.

To this aim, we tested three groups: patients with PD off levodopa medication, patients with PD on levodopa medication, and an unmedicated control group with polyneuropathies (peripheral nerve damage). By combining behavioral and computational analyses, we isolate deficits specific to dopamine depletion in PD, evaluate the restorative effects of levodopa medication, and assess the extent to which medicated patients resemble controls.

## RESULTS

We compared patients with PD on and off medication and age-matched controls in their ability to perform a spatial reward learning task (Table 1). All patients with PD (*n* = 68) regularly received dopaminergic medication (levodopa) for symptomatic treatment and were randomly assigned to be tested either after taking their regular dose (PD+; *n* = 34) or in a state of dopaminergic depletion shortly before their next dose (PD−; *n* = 34). The control group consisted of age-matched patients with polyneuropathies, i.e., disorders or damage of the peripheral nerves without involvement of the central nervous system (*n* = 35).

**Table 1.** Descriptive characteristics. Shown are means (SD); *P* values pertain to *X*^2^ test for gender and one-way ANOVAs for the other variables. BDI-II, Beck Depression Inventory II (*59*); HY, Hoehn-Yahr scale (*57*); MMSE, mini-mental state examination (*58*); levodopa EDD, Levodopa equivalent daily dose, calculated according to (*69*), for patients who consented to the access of their medical records (14 PD− and 12 PD+ patients).

	PD−	PD+	Control	*P*
*n*	34	34	35	
Gender (% female)	15 (44%)	18 (53%)	21 (60%)	0.4
Age (years)	67.0 (6.5)	64.3 (6.2)	65.5 (7.0)	0.2
Depression (BDI-II)	8.6 (4.1)	8.6 (3.6)	8.5 (3.4)	>0.9
Cognitive functioning (MMSE)	28.8 (0.8)	29.1 (0.8)	28.8 (0.9)	0.3
PD severity (HY)	1.9 (0.7)	1.9 (0.6)		0.9
Levodopa EDD (mg/day)	809.0 (230.8)	894.0 (441.7)		0.6
Time since medication (min)	248.5 (32.7)	104.4 (59.2)		<0.001

All three groups were of interest to our investigation. The comparison between PD− patients off medication and the control group indicates deficits specific to PD in a state of (natural) dopamine depletion. The comparison between patients with PD off (PD−) and on medication (PD+) shows to what degree levodopa improves performance within PD. Last, comparing PD+ patients with controls provides an estimate of the degree to which levodopa compensates for dopamine-related deficits in behavior.

### Behavioral results

**Fig. 1.**
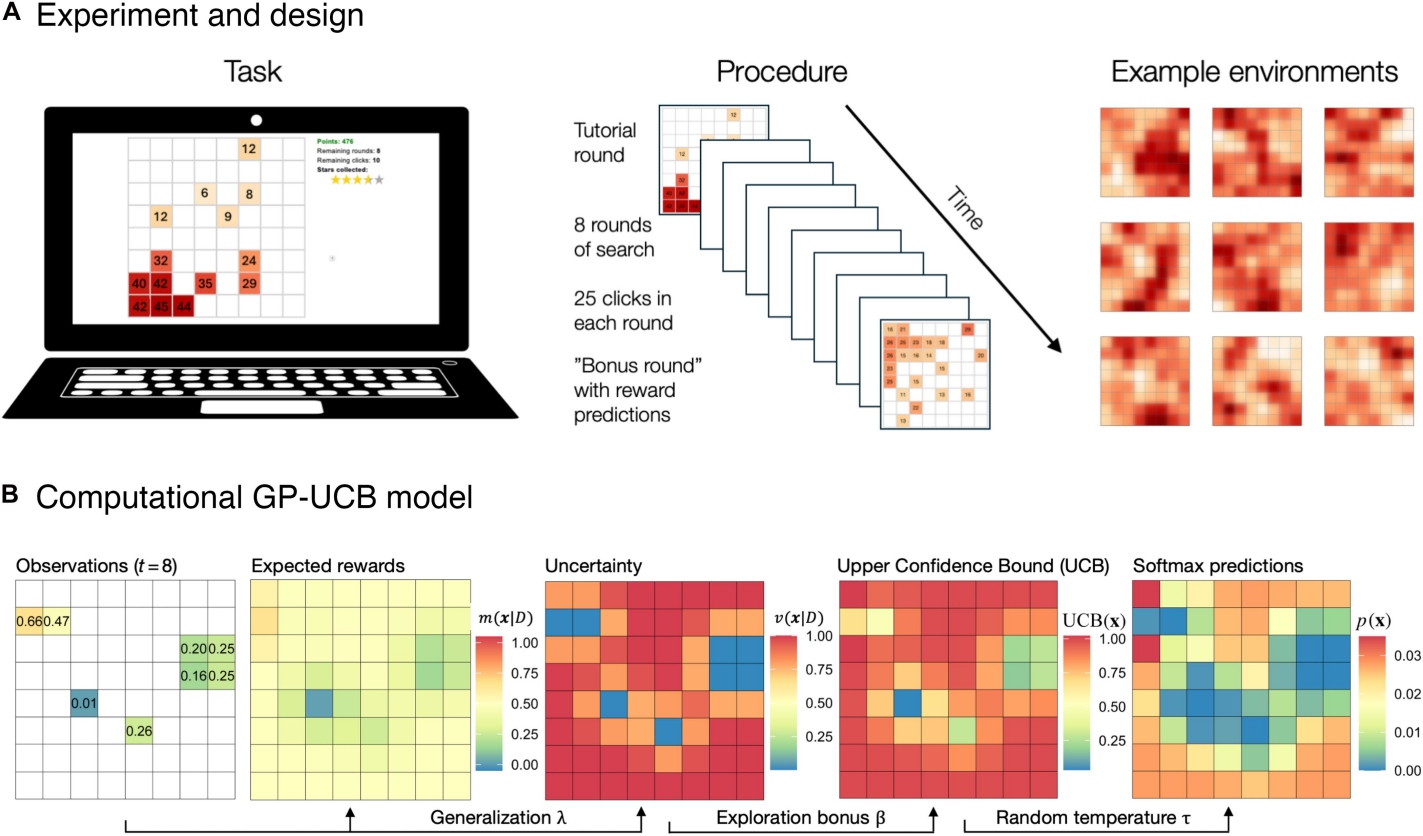
Spatially correlated multi-armed bandit task and computational model. (**A**) Schematic depiction of the task, illustrating example choices on the 8 by 8 grid that participants used to accumulate rewards. Rewards were shown numerically and mapped onto nine shades of red across the reward range, with darker shades corresponding to higher rewards. Each participant completed 10 rounds, where in each round a new environments was presented. The first round was a tutorial round and the last round was a bonus round where participants made predictions for five randomly selected tiles after making 15 explore-exploit choices. In each round, a new environment was used, drawn randomly from a set of 40 environments with the same level of spatial correlation. The right panel shows nine example environments. All displayed grids are illustrative and do not represent complete or specific rounds. (**B**) Task behavior was modeled using a Gaussian process (GP) with upper confidence bound (UCB) sampling. The GP-UCB model combines similarity-based generalization with mechanisms for uncertainty-directed and random exploration. First, the observed data are used to fit a GP model, which estimates the expected rewards *m*(**x**) and associated uncertainty *v*(**x**) for each option (tile), conditional on the observed data. The amount of generalization is determined by parameter λ, the length-scale of the radial basis function (RBF) kernel, which in the GP determines how quickly reward correlations decay with spatial distance. Next, options are valued using UCB sampling, which inflates reward expectations with an uncertainty bonus β, representing the extent of uncertainty-directed exploration. Last, UCB values are passed through a softmax choice rule, with the temperature parameter τ reflecting the degree of random exploration. (B) is reproduced from (*37*) under the terms of the Creative Commons Attribution 4.0 International License [CC BY 4.0 (https://creativecommons.org/licenses/by/4.0/deed.en)].

Participants accumulated rewards by selecting tiles (options) on a 8 by 8 grid where the reward for each tile was drawn from a Gaussian distribution (Fig. 1). The spatial correlation between tiles could be used to optimize search behavior and efficiently navigate the exploration-exploitation trade-off. We analyzed behavior in terms of performance, temporal dynamics of exploration behavior, and spatial trajectories of search, based on eight rounds of 25 trials per participant.

### Impaired reward learning in patients with PD off medication

The key performance measure was the mean reward earned. Hierarchical regression analyses revealed an effect of group on mean reward earned, but no effects of game round, PD severity, depressive symptoms, or cognitive functioning (Supplementary Materials; tables S1 to S3). These variables were therefore not considered in subsequent analyses. We also analyzed decision latencies (i.e., response time between consecutive clicks on the grid), but did not observe a difference between patients with PD on and off medication (Supplementary Materials).

**Fig. 2.**
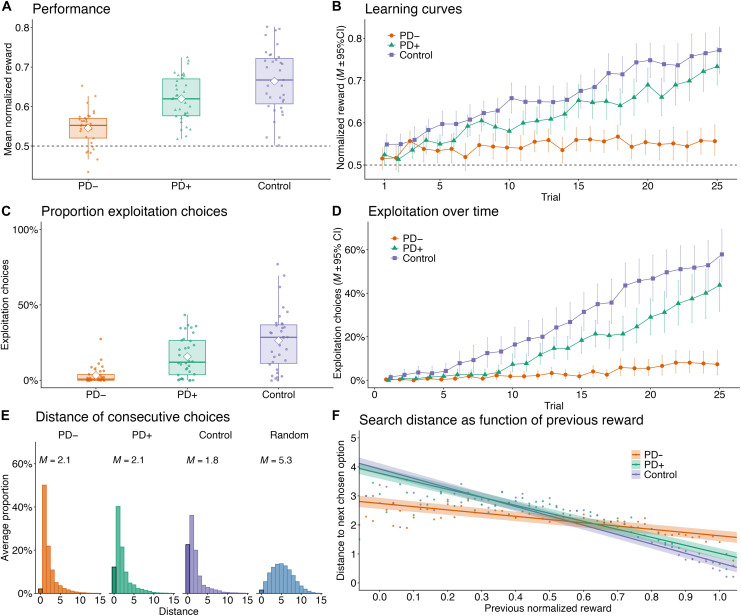
Behavioral results. (**A**) Mean reward earned by group. Each dot is one participants’ mean reward across all rounds and trials. Across all figures, box plots indicate the median (horizontal bar) the interquartile range (box) and the mean (white diamond). Whiskers extend from the box to 1.5 × interquartile range. The black and dotted horizontal line indicates random performance. (**B**) Learning curves, showing mean reward earned on each trial, averaged across rounds. Error bars for learning curves indicated 95% confidence interval (CI). (**C**) Mean proportions of exploitation decisions, aggregated over trials and rounds. Each dot is one participant. An exploitative choice is defined as re-clicking a previously revealed tile. (**D**) Mean proportion of exploitation decisions per trial, averaged over rounds. (**E**) Manhattan distance among consecutive clicks. A repeat click has a distance of zero (marked by a black outline); clicks on neighboring tiles have a distance of 1, and distances >1 correspond to clicks further away from the previous click. The right-most panel shows the distribution of distances for a random learner that selects in each trial with uniform probability among all options. (**F**) Predictions of a Bayesian hierarchical regression with search distance as a function of reward earned on the previous trial. Dots are the empirical mean distances for each reward value, averaged over participants, trials, and rounds.

Figure 2A shows the mean reward earned across all trials. PD+ patients on medication achieved substantially higher rewards than PD− patients off medication [*t*(66) = 5.9, *P* < 0.001, *d* = 1.4, Bayes factor (BF) > 100], indicating a strong beneficial effect of levodopa. Controls achieved slightly higher rewards than PD+ [*t*(67) = 2.6, *P* = 0.01, *d* = 0.6, and BF = 4.5] and were much better than PD− patients [*t*(67) = 7.5, *P* < 0.001, *d* = 1.8, and BF > 100].

The stark performance disadvantage for PD− gradually developed across trials (Fig. 2B). While controls and PD+ showed relatively steep learning curves, the learning slope of PD− patients was barely positive, suggesting a substantial deficit in managing the exploration-exploitation trade-off.

### Levodopa mitigates deficits in exploration and exploitation

We next analyzed the trade-off between exploration and exploitation. Figure 2C shows that differences in reward accumulation are driven by learners’ ability to adequately balance exploration (selecting novel options) and exploitation (re-clicking tiles). PD+ patients exploited substantially more than PD− patients [3%versus16%,t(66)=5.0, P<0.001, d=1.2, and BF>100]. Controls exploited even more than PD+ patients, although this effect was weaker [16%versus26%,t(67)=2.6, P=0.01, d=0.6, and BF=4.3].

Mirroring the reward learning curves, controls and PD+ patients shifted more efficiently from exploring novel options to exploiting known options over time (Fig. 2D). Controls began exploiting earlier in the round and exhibited a stronger overall tendency toward exploitation compared to PD+ patients, explaining their relative performance advantage. In stark contrast, PD− patients predominantly engaged in exploration, as reflected in their low proportion of exploit choices (i.e., re-clicking tiles), and showed only a weak tendency toward exploitation as the search horizon approached its end.

Consistent with these observations, nearly all controls (94%) and PD+ patients (88%) made at least one exploit choice, whereas only 65% of PD− patients did. Among participants who engaged in each choice type, PD+ patients earned higher mean rewards than PD− patients during both exploration [t(50)=2.5, P=0.016, d=0.7, and BF=3.3] and exploitation [t(50)=3.6, P<0.001, d=1.0, and BF=38]. Thus, when they exploited and explored, PD+ patients on medication showed higher efficiency in both behaviors. Similarly, controls earned higher rewards than PD− patients during exploration [t(53)=3.0, P=0.004, d=0.8, and BF=10] and exploitation [t(53)=3.9, P<0.001, d=1.1, and BF=83]. Controls and PD+ patients did not differ (both P>0.27), indicating that the efficiency of exploration and exploitation was largely restored by levodopa medication.

### Levodopa restores reward sensitivity during exploration

We analyzed the spatial trajectory of individuals’ search processes by quantifying step-by-step distances and testing how obtained rewards shaped subsequent choices. Figure 2E shows the distribution of distances among consecutive clicks, illustrating how participants explored the space. In all groups, the most frequent choice was to click on a directly neighboring tile (distance = 1), indicating a localized search strategy. This locality bias was strongest in PD− patients off medication, who most frequently selected directly neighboring tiles. Across all choices, controls had lower search distances than both patients off medication [t(67)=−2.6, P=0.011, d=0.6, and BF=4.2] and on medication [t(67)=−2.3, P=0.023, d=0.6, and BF=2.4]. The mean distance of the two PD groups did not differ (P>0.7), but the distributions indicate different underlying strategies: fewer repeats (distance 0) but more near choices (distance 1) in PD− patients, balancing out against PD+ patients who made more exploit choices but clicked less often on directly neighboring tiles. Simulations of a random learner show a very different pattern compared to human behavior (Fig. 2E, right panel), indicating that all groups deviated substantially from purely random choice behavior.

To better understand when participants chose to search locally versus farther away, we next examined how search distances depended on the reward obtained from the previous choice (Fig. 2F). If a large reward was obtained, learners should search more locally; if a low reward was obtained, learners should search farther away. This behavior was observed in both controls and PD patients on medication, showing how they leveraged the structure of the environment to guide their search process. In contrast, the exploration strategy for PD patients off medication was largely insensitive to reward magnitude, indicating a deficit in goal-directed exploration based on the structural regularities of the environment.

### The Gaussian process upper confidence bound model captures key behavioral aspects of exploration and generalization

The behavioral analyses showed that individuals in a dopamine-depleted state exhibit a severe deficit in balancing exploration and exploitation. By contrast, the behavior of patients on medication was markedly improved and largely resembled controls. The increased exploration in patients off medication could reflect more random choice behavior, an increased emphasis on uncertainty-directed exploration, or a deficit in using the structural regularities of the grid (i.e., impaired generalization).

To disentangle these mechanisms, we used the Gaussian process upper confidence bound (GP-UCB) model (Fig. 1B; see Materials and Methods for formal specification). The model integrates similarity-based generalization with two distinct exploration mechanisms: uncertainty-directed exploration, which seeks to reduce uncertainty about rewards, and random exploration, which adds stochastic noise without being directed towards a particular goal (*29*, *34*). These processes are captured by three key parameters: the generalization parameter λ (Eq. 2), which determines how strongly rewards are generalized across options; the uncertainty bonus β (Eq. 6), which governs the degree of uncertainty-directed exploration by determining the value given to uncertainty; and the temperature parameter τ (Eq. 7), which captures random exploration. Behaviorally, these correspond to how broadly participants generalized from observed rewards (λ), how strongly they prioritized uncertain options (β), and how variable their choices were independently of option value (τ).

In previous studies using the same experimental paradigm, the GP-UCB model provided the best account of exploratory behavior in healthy participants (*29*, *35*–*37*, *39*, *40*). By decomposing exploration into generalization (λ), uncertainty-driven exploration (β), and random exploration (τ), the model allows us to identify which mechanisms are altered by PD and medication. In doing so, it directly connects to prior findings that levodopa impairs discrimination learning while sparing generalization in PD (*41*), that levodopa reduces directed exploration in healthy participants (*26*), and that PD disrupts the overall exploration-exploitation balance (*14*–*16*).

### All components of the GP-UCB model are critical to explain behavior

To assess the contribution of each model component (generalization, uncertainty-directed exploration, and random exploration), we compared the predictive accuracy of the GP-UCB model to variants where we lesion away each component (Materials and Methods). The λ lesion model removes the ability to learn about the spatial regularities on the grid and thus to generalize. Instead, it is assumed that all options are learned independently [i.e., Bayesian mean tracker (BMT); Eqs. 9 to 11]. The β lesion model disregards uncertainty and values options solely based on their reward expectations, corresponding to a mean greedy sampling strategy (i.e., β=0 in Eq. 6). Last, the τ lesion model replaces the softmax choice function (Eq. 7) with an ϵ-greedy policy (Eq. 12) as an alternative mechanism for random exploration, which samples uniformly from all options with fixed probability ϵ, otherwise selecting the option with the highest UCB value.

**Fig. 3.**
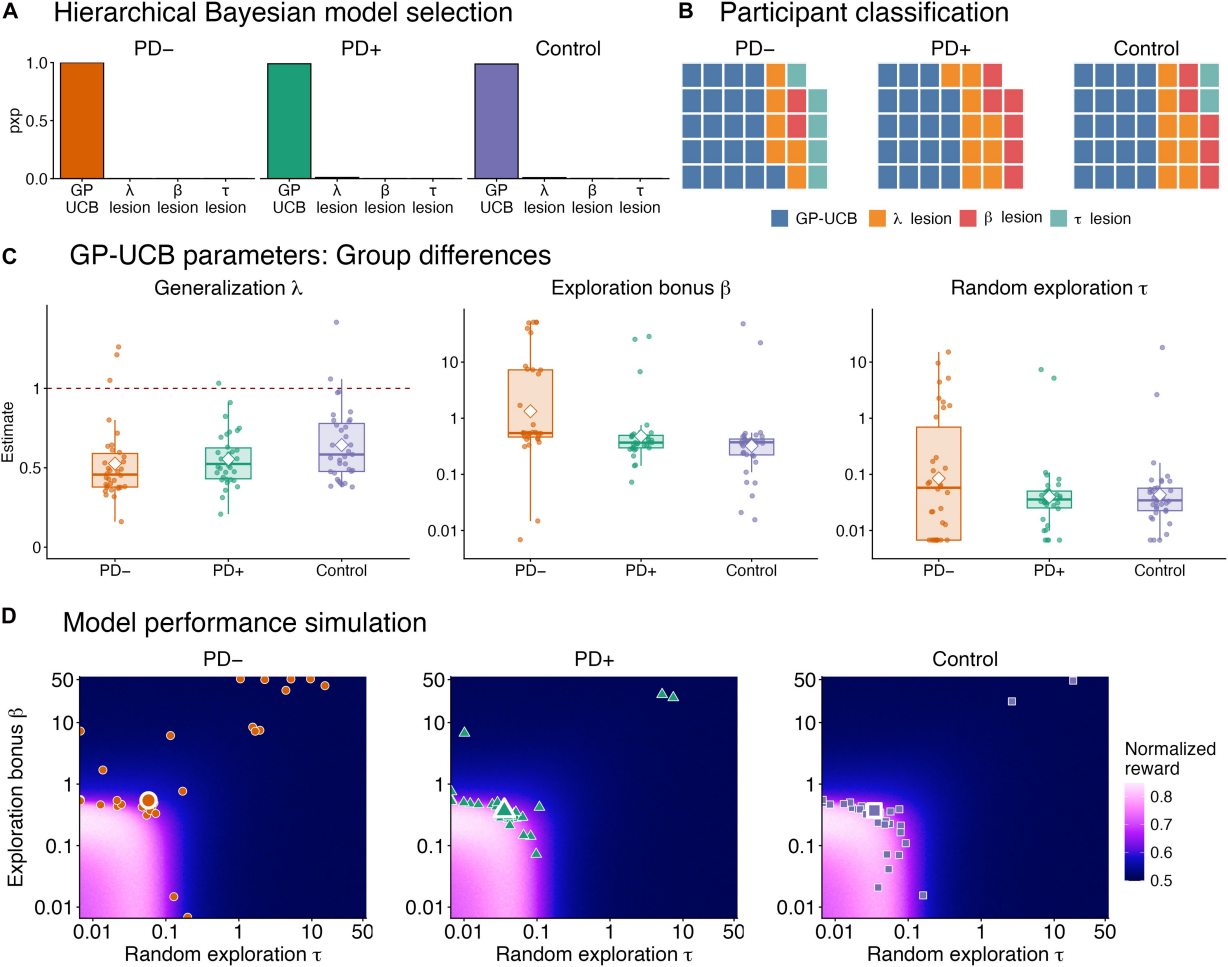
Computational modeling results. (**A**) Hierarchical Bayesian model comparison based on the protected exceedance probability (pxp) estimating which model is more frequent in the population. In each group, the GP-UCB model was the most likely model. (**B**) Each participant was assigned to the model that best described their behavior (highest *R*^2^ or, equivalently, lowest cross-validated log loss). In each group, the GP-UCB model accounted for the highest proportion of learners. (**C**) Estimates for the generalization parameter λ, the uncertainty-directed exploration parameter β, and the random exploration parameter τ. The red dashed line in the λ plot denotes the true amount of spatial correlation (λ=1). (**D**) Performance landscape of the GP-UCB model across different amounts of random exploration τ and uncertainty-directed exploration β. The amount of generalization was fixed to λ=1, the true amount of correlation in the environment, and β and τ were varied, with 1000 simulated learners per parameter combination. Each dot shows one participant; the larger markers indicate the group median.

The predictive accuracy of models was assessed through leave-one-round-out cross-validation, where predictive performance was quantified by the predicted choice probabilities and log loss across all out-of-sample predictions. For group-level model selection, we computed protected exceedance probabilities (pxp), which quantify the probability that a given model is more frequent in the population than all competing models (*42*). Across all three groups, the GP-UCB model outperformed all lesioned models, suggesting it as the as the most frequent population model (Fig. 3A).

In addition, we used a pseudo-*R*^2^ measure (Eq. 13) to quantify predictive accuracy, where $R^{2}=0$ corresponds to the accuracy at chance level and $R^{2}=1$ corresponds to perfect accuracy. Consistent with the more diffuse exploratory behavior in PD−, absolute predictive accuracy was lower in the PD− group than in PD+ patients and controls (fig. S5), because exact trial-by-trial choices were inherently harder to predict. However, across groups and in line with the pxp analysis, the GP-UCB model outperformed each of the lesioned models (*t*-tests; all P<0.01, all BF≥12; table S6). In each group, the GP-UCB model was also the most predictive (highest *R*^2^) model for the majority of participants (Fig. 3B).

Overall, these results show that all three components of the GP-UCB model are critical for predicting behavior, aligning with findings from previous studies covering a broad age range in nonclinical populations (*29*, *35*–*37*, *39*).

### Levodopa selectively alters uncertainty-directed but not random exploration in PD

The parameters of the GP-UCB model provide model-based indices of distinct facets of learning and exploration. The parameter λ of the learning model reflects how strongly a learner generalizes, the uncertainty bonus β tracks the level of uncertainty-directed exploration, and the temperature parameter τ indicates the amount of random exploration (Fig. 3C).

Compared to controls, PD− patients had a significantly reduced generalization parameter λ, indicating a deficit in learning and utilizing the spatial regularities of the grid (U=832, P=0.004, $r_{\tau }=0.28$, and BF=6.0). PD+ patients tended to generalize more than PD− patients and less than controls, but the differences were not statistically significant (P=.12 and BF=0.47).

The size of the uncertainty bonus β markedly differed between groups, with PD− patients showing the highest levels and substantial variability compared to the other groups (Fig. 3B). Both PD+ patients (U=271, P<0.001, $r_{\tau }=-0.38$, and BF=19) and controls (U=224, P<0.001, $r_{\tau }=-0.44$, BF>100) had lower uncertainty bonuses than patients off medication. PD+ patients and controls did not differ.

We did not observe mean differences in the temperature parameter τ (all P>0.46) among groups, indicating comparable average levels of random exploration across groups. However, variability was markedly higher in the PD− group than in PD+ patients and controls, similar to the pattern observed for the uncertainty bonus β. Together, these analyses suggest that the effects of dopaminergic medication are specifically reflected in the degree of uncertainty-directed exploration, with levodopa regulating the “exploration bonus” to levels comparable to those observed in controls without PD. This aligns with findings from a restless bandit paradigm with healthy volunteers, where levodopa reduced the amount of directed exploration, while random exploration remained unaffected by medication (*26*).

### Controls and patients with PD on medication balance directed and undirected exploration better than patients with PD off medication

To evaluate how well different parameter settings balance exploration and exploitation, we conducted simulations with the GP-UCB model. In these simulations, we set the amount of generalization to λ=1, matching the true smoothness of the reward function in the environments, and systematically varied the amount of random exploration (τ) and the size of the uncertainty bonus (β) to simulate the performance of the GP-UCB model under different levels of directed and random exploration. Figure 3D depicts the resulting performance landscape together with the inferred parameters of all participants. Compared to PD− patients off medication, the parameter estimates for PD+ patients on medication and controls cluster more closely around the optimal region. Particularly the inflated exploration bonus β places PD− patients in regions yielding low performance.

## DISCUSSION

We investigated reward learning and exploratory behavior of PD patients on medication (PD+), PD patients off medication (PD−) and controls in a structured reward-learning task with a large decision space. Because rewards were spatially correlated, learners had to learn and leverage hidden structure to generalize to novel options, which placed specific demands on goal-directed exploration. PD− patients struggled to exploit known high-value options and showed little sensitivity to previous rewards during exploration. A computational model separating random from uncertainty-directed exploration revealed that PD− patients tended towards excessive uncertainty-directed exploration, suggesting impaired regulation of exploratory behavior in a dopamine-depleted state. The performance deficits align with prior evidence of impaired reward learning in PD (*7*, *8*, *11*–*14*, *43*–*45*), although in our task the impairments were more severe. This suggests that structured tasks that require learning about latent regularities are particularly affected in PD, highlighting their potential diagnostic value.

On medication, patients with PD largely resembled controls, showing how dopaminergic medication regulates the balance between exploration and exploitation. The restorative effect of levodopa suggests that the observed deficits were specifically caused by dopamine depletion and not by other mechanisms underlying PD such as a loss of noradrenergic neurons or neuroinflammatory processes. Notably, while other pharmacological studies in PD (*7*, *13*, *45*) also show beneficial effects of levodopa, the improvements in the present study were much stronger.

Behaviorally, the performance deficits of PD− patients result from too much exploration and too little exploitation, consistent with findings in patients with PD and apathy (*14*). Notably, failures to exploit in our task cannot be attributed to working memory, as previous rewards remained visible. The computational analyses provided additional insight, as goal-directed exploration in the present task reflects two factors: using the spatial correlation in the environment and the consideration of uncertainty in the valuation of choice options. While random exploration was comparable across groups, both generalization and uncertainty-directed exploration were impaired in PD− patients.

First, deficits in generalization were evident in how PD− patients adjusted their search distances based on the reward magnitude of the preceding choice. Leveraging the spatial correlation of rewards should promote local search after high rewards and more distant search after low rewards. Yet, PD− showed only minimal adaption to this structure, which, computationally, resulted in a reduced generalization parameter λ. These results are consistent with a general executive planning deficit in PD (*46*), and with impairments in model-based learning (*10*). Second, we observed a strong increase in the valuation of uncertainty, such that PD− patients placed an overly strong emphasis on uncertainty-directed exploration. The observed increase in uncertainty-guided exploration may seem surprising, as dopamine is often linked to novelty seeking (*47*). However, our findings align with recent findings that levodopa in healthy participants attenuated uncertainty-directed exploration in a restless bandit task (*26*), and that in monkeys a pharmacological inhibition of the dopamine transporter increased their preference for novel options (*48*). This combination of lowered reward sensitivity and heightened exploration resembles mechanisms discussed in addiction, where low tonic dopamine is argued to drive drug-seeking to offset reduced reward sensitivity [the dopamine hypothesis of drug addiction (*49*)]. Analogously, low tonic dopamine in PD may diminish the salience of known rewards and induce a tendency towards exploring novel or uncertain options. This parallel suggests that a dopamine-depleted state can drive maladaptive shifts in behavioral strategies across distinct clinical conditions.

Our study design differed from previous work in two relevant aspects. First, instead of a healthy control group, we included patients with polyneuropathies. While this control group is not expected to exhibit specific deficits in reward learning or exploration, it limits our ability to conclude that levodopa fully restores performance to the level of healthy individuals. At the same time, an advantage of our non-healthy control group is that group differences are less likely to be attributable to nonspecific effects of “having a disease.” All three groups in our study were closely matched on age, gender, depressive symptoms, and also in basic cognitive functioning, which did not differ between patients with PD tested on and off medication. Second, our withdrawal period in the PD− group was shorter than in most previous studies, which often use a full overnight withdrawal [e.g., (*50*, *51*)]. This limitation arose from the outpatient setting, where minimizing disruption to the patients’ regular treatment was a priority. A within-subject crossover design, in which the same patients are tested both on and off medication in counterbalanced order, would provide stronger causal evidence regarding medication effects, but was not possible to implement in the present clinical context. Nevertheless, the robust behavioral effects observed suggest that the shorter withdrawal period did not substantially affect our results. Future studies could complement the present paradigm with additional motor measures, such as erroneous clicks or movement trajectories, as well as standardized assessments of disease severity on and off medication. This would allow a more fine-grained characterization of motor and medication-related influences on behavior.

In sum, our results point to a crucial role of dopamine in the regulation of goal-directed exploration. Conversely, the loss of such regulatory dopaminergic function in PD may account for the marked deficits in reward learning, set shifting (*52*) and other instrumental activities of daily living that require intact exploratory behavior, such as shopping, transportation, and money management (*53*–*56*). Dopaminergic medication might be an effective therapy in PD to mitigate these impairments, helping patients to navigate the ubiquitous explore-exploit trade-offs they encounter in their daily lives.

## MATERIALS AND METHODS

### Experimental design

Participants performed a reward-based decision-making task based on a spatially correlated multi-armed bandit task (Fig. 1A). Participants completed 10 rounds of the task, each featuring a new environment with the same level of spatial correlation. At the start of each round, one tile was randomly revealed, and participants sequentially sampled 25 tiles. On each trial, they could choose to either click a new tile (explore) or re-click a previously selected tile (exploit). Selections were made by selecting the tile on the computer screen using a mouse, upon which they received a reward in the range [0,50]. Re-clicked tiles showed small variations in reward due to normally distributed noise. Rewards were shown numerically together with one of nine corresponding shades of red spanning the displayed reward range, with darker shades of red indicating higher rewards.

The environment in each round was sampled from a pool of 40 distinct environments, which were generated using a radial basis function kernel with length-scale parameter λ=1, creating a bivariate reward function on the grid that maps each tile location to a specific reward value. These reward functions gradually varied across the grid, creating environments with spatially correlated rewards (Fig. 1B, right panel). Expressed as a Pearson correlation, the rewards of directly neighboring options correlated at approximately r≈0.6, decreasing exponentially with spatial distance.

In each of 10 rounds, participants had 25 choices to accumulate rewards. The first round served as a tutorial to familiarize participants with the task, goal, spatial correlation of rewards, possibility of re-clicking tiles, and the length of the search horizon. Before the actual task started, each participant had to pass an instruction check with questions pertaining to the instructed goal, that points could be collected both by revealing new tiles and by re-clicking previously revealed tiles, and that points tended to cluster. Data from the tutorial round was excluded from the analyses. The 10th and final round was a bonus round where, after 15 choices, participants were asked to predict rewards for five unrevealed options. Data from this round were also excluded from the main analysis and analyzed separately (Supplementary Materials).

### Sample

We tested *n* = 66 adult participants with PD who regularly receive levodopa (levodopa) for symptomatic treatment. Participants were recruited and tested at a neurologist’s outpatient practice; sessions lasted 30 to 45 min. Eligible individuals diagnosed with PD were evaluated based on the Hoehn-Yahr scores recorded in their patient files. The scale assesses disease severity and motor impairments based on a score from 1 to 5, with higher scores indicating greater severity (*57*). Recruitment was limited to individuals with scores between 1 and 3, and patients who did not receive antipsychotic medication (except for one patient who received a small dose of 25 mg of clozapine to counteract delusional side effects of levodopa).

The study was approved by the Institutional Review Board of the Health and Medical University, Potsdam, Germany (approval nos. HMU2024-06 and HMU2024-06A). All procedures were carried out in accordance with the Declaration of Helsinki and applicable institutional and national guidelines and regulations. All participants provided written informed consent.

Patients with PD were randomly assigned to two conditions: testing on medication (PD+) and off medication (PD−). In the PD+ group (*n* = 33), patients’ scheduled levodopa dose was administered at least 30 min before the start of the experiment. One patient was treated with continuous subcutaneous infusion pump and was therefore assigned to the PD+ group. In the PD− group (*n* = 33), participants were tested immediately before their next scheduled dose, when dopaminergic stimulation from medication was presumably minimal. In sum, the PD+ “on medication” group was tested just after taking levodopa and patients in the PD− “off medication” group were tested just before their next scheduled dose. The behavioral data of one patient in the PD− group was lost due to a computer crash and excluded from the analysis.

The control group (*n* = 35) was recruited in the same practice and consisted of individuals of similar age diagnosed with polyneuropathies, a condition affecting the peripheral nervous system that can lead to physical symptoms such as pain, sensory loss, or motor weakness. However, unlike PD, polyneuropathies typically do not involve central dopaminergic dysfunction or cognitive impairment.

### Clinical assessment

We used standardized measures assessing PD severity, basic cognitive function, and depressive symptoms. PD severity was evaluated using the Hoehn-Yahr scale as documented in patients’ most recent clinical records, which rates motor impairments such as postural instability and gait difficulties (*57*). Basic cognitive function was assessed through the Mini-Mental State Examination (MMSE) (*58*). The test comprises 30 questions pertaining to different domains, including memory (e.g., recalling three objects), temporal and spatial orientation (e.g., date and location), and arithmetic ability. Last, all participants answered the German version of the Beck Depression Inventory II, a self-report inventory measuring depressive symptoms (*59*, *60*). No differences on any of these measures was found (Table 1).

### Statistical analysis

Neither round number across the eight analyzed search rounds nor any clinical markers of PD (depressive symptoms, cognitive screening, and PD severity) affected performance (tables S1 to S3). Therefore, these variables were not further considered in the analyses. Data analysis comprised group comparisons, hierarchical regression and correlation analyses. We report frequentist statistics as well as BFs to quantify the relative evidence of the alternative hypothesis (*H*_1_) over the null hypothesis (*H*_0_) (see the Supplementary Materials for details). To analyze the effect of previous reward on search distance we performed a Bayesian hierarchical regression, including fixed effects for reward, group, and the interaction reward × group, as well as subject-wise random intercepts.

### Computational modeling

#### GP-UCB model

The GP-UCB model combines a Bayesian framework for value generalization (GP) with a combination of uncertainty-directed and random exploration (UCB). As such, it provides a comprehensive computational framework for exploratory behavior and how this behavior is shaped by mental health conditions and medication.

#### Gaussian process generalization

To model how participants generalize observed rewards across the two-dimensional grid, we use GP regression (*61*). A GP is a Bayesian nonparametric model of function learning that has been used as a psychological model of human generalization (*34*) across spatial (*29*, *37*), abstract (*39*), social (*32*, *40*), and graph-structured domains (*62*), predicting both choices and judgments about reward expectations and confidence. Formally, a GP defines a probability distribution over functions mapping inputs to outputs f:X→Y. In our case, these functions map grid locations x∈X to scalar reward observations y∈Y, with the prior distribution taking the form of a multivariate Gaussian

$$f∼\text{GP}\left[m\left(x\right),k\left(x,x^{\prime }\right)\right]$$ (1)

The GP is fully specified by prior mean function m(x) defining the prior expectations of each input, and a kernel (covariance) $k\left(x,x^{\prime }\right)$ encoding how strongly rewards at two locations are expected to covary as a function of their distance (see Eq. 2). Without loss of generality, we set the prior mean to zero (*61*) and use the common RBF kernel

$$k\left(x,x^{\prime }\right)=\text{exp}\left(-\frac{{\left\|x-x^{\prime }\right\|}^{2}}{2\lambda^{2}}\right)$$ (2)

Here, x and $x^{\prime }$ are the coordinates of two tiles on the grid, and λ is the length-scale parameter, which governs the amount of generalization (i.e., the smoothness of the function). Higher values of λ imply smoother functions, leading to stronger expectations regarding reward correlations. Lower values of λ entail rougher functions, i.e., less correlation among similar options. In our analyses, we treat λ as a free parameter representing the extent to which learners generalize rewards as function of spatial proximity.

To compute posterior predictions for any target location $x_{⋆}$, we condition the model on a set of observations $D_{t}=\left\{X_{t},y_{t}\right\}$ of choices $X_{t}=\left[x_{1},\ldots x_{t}\right]$ and corresponding reward observations $y_{t}=\left[y_{1},\ldots ,y_{t}\right]$ at time *t*. This posterior also takes the form of a multivariate Gaussian

$$f\left(x_{⋆}\right)|D_{t}∼N\left[m\left(x_{⋆}|D_{t}\right),v\left(x_{⋆}|D_{t}\right)\right]$$ (3)

which is entirely defined by a posterior mean $m\left(x_{⋆}|D_{t}\right)$ and a posterior variance $v\left(x_{⋆}|D_{t}\right)$. These are computed as

$$m\left(x_{⋆}|D_{t}\right)=k_{⋆}{\left[K_{X,X}+\sigma_{\epsilon }^{2}I\right]}^{-1}y_{t}$$ (4)

$$v\left(x_{⋆}|D_{t}\right)=k\left(x_{⋆},x_{⋆}\right)-k_{⋆}^{⊤}{\left[K_{X,X}+\sigma_{\epsilon }^{2}I\right]}^{-1}k_{⋆}$$ (5)

Here, $k_{⋆}=\left[k\left(x_{1},x_{⋆}\right),\ldots ,k\left(x_{t},x_{⋆}\right)\right]$ is the vector of kernel similarities between past observations and the target location, $K_{X,X}$ is a matrix of pairwise kernel similarities between all past observations in $X_{t}$, I is a t×t identity matrix, and $\sigma_{\epsilon }^{2}$ is the observation noise capturing the stochasticity of reward observations and is fixed to the true reward variability of each arm of the bandit $\sigma_{\epsilon }^{2}=.0001$.

#### UCB sampling

UCB sampling considers both reward estimates and their uncertainty when valuing options. Formally, this is done by adding an uncertainty bonus to the expected rewards of each option

$$\text{UCB}\left(x\right)=m\left(x|D_{t}\right)+\beta \sqrt{v\left(x\;|\;D_{t}\right)}$$ (6)

where the expected reward of an option $m\left(x\;|\;D_{t}\right)$ captures its exploitation value, and the scaled uncertainty $\beta \sqrt{v\left(x\;|\;D_{t}\right)}$ captures its exploration value, with β modulating how much exploration is promoted relative to exploitation.

Balancing rewards and uncertainty requires an appropriate value for β. If β is too small, the learner fails to explore promising but uncertain options, leading to a disproportionate focus on exploitation. Conversely, if β is too large, the uncertainty bonus overrules any reward differences, making all options appear equally attractive and leading to overexploration.

#### Softmax choice rule

The final step of the model is to translate UCB values into choice probabilities, describing how likely an agent will select each of the 64 options. This is implemented using a softmax function

$$p\left(x\right)=\frac{\text{exp}\text{[UCB}\left(x\right)/\tau ]}{\sum_{j=1}^{N}\text{exp}\text{[UCB}\left(x_{j}\right)/\tau ]}$$ (7)

The amount of randomness in the choice probabilities is governed by the temperature parameter τ. Higher values of τ make the choice probabilities more uniform, such that the choice behavior is less influenced by options’ UCB values and more random. Lower value of τ imply that the learner is more sensitive to options’ UCB values, making them increasingly likely to be selected. In the limits, if τ→0, choice behavior reduces to a greedy mean policy that always selects the option with the highest value (pure exploitation), and if τ→∞, all options are chosen with equal probability (pure exploration). Here, we treat the temperature parameter τ as a computational marker of a learner’s tendency to explore randomly, i.e., in an undirected fashion through inherent decision noise.

### Lesioned models

To establish that all components of the GP-UCB model are required to explain behavior, we implemented three lesion variants of the model (*37*).

The λ lesion model removes the ability to generalize, such that options’ rewards are learned independently via a BMT. The BMT is a Kalman filter with time-invariant rewards (*63*, *64*), and as such, can be interpreted as Bayesian variant (*65*) of the classic Rescorla-Wagner (*66*) or Q-learning models (*67*). Intuitively, reward estimates are updated as a function of prediction error, where the learning rate is dynamically defined based on the degree of uncertainty of the model.

Like the GP, the BMT also assumes a Gaussian prior distribution of reward expectations, but does so independently for each option **x**

$$p\left[r_{0}\left(x\right)\right]∼N\left[m_{0}\left(x\right),v_{0}\left(x\right)\right]$$ (8)

where $m_{0}\left(x\right)=0$ as in the GP, and we set $v_{0}\left(x\right)=5$ following (*37*).

The BMT then computes a posterior distribution of the expected reward for each option, also in the form of a Gaussian, but where the posterior mean $m_{t}\left(x\right)$ and posterior variance $v_{t}\left(x\right)$ are defined independently for each option and computed by the following updates

$$m_{t+1}\left(x\right)=m_{t}\left(x\right)+\delta_{t}\left(x\right)G_{t}\left(x\right)\left[y_{t}\left(x\right)-m_{t}\left(x\right)\right]$$ (9)

$$v_{t+1}\left(x\right)=v_{t}\left(x\right)\left[1-\delta_{t}\left(x\right)G_{t}\left(x\right)\right]$$ (10)

Both updates use $\delta_{t}\left(x\right)=1$ if option **x** was chosen on trial *t*, and $\delta_{t}\left(x\right)=0$ otherwise. Thus, the posterior mean and variance are only updated for the chosen option. The update of the mean is based on the prediction error $y_{t}\left(x\right)-m_{t}\left(x\right)$ between observed and anticipated reward, while the magnitude of the update is based on the Kalman gain $G_{t}\left(x\right)$

$$G_{t}\left(x\right)=\frac{v_{t}\left(x\right)}{v_{t}\left(x\right)+\theta_{\epsilon }^{2}}$$ (11)

analogous to the learning rate of the Rescorla-Wagner or Q-learning models. Here, the Kalman gain is dynamically defined as a ratio of variance terms, where $v_{t}\left(x\right)$ is the posterior variance estimate and $\theta_{\epsilon }^{2}$ is the error variance, which we treat as a free parameter and can be interpreted as an inverse sensitivity parameter. Smaller values of $\theta_{\epsilon }^{2}$ thus result in larger updates of the mean.

The β lesion model evaluates options solely based on their expected rewards, corresponding to a mean-greedy sampling strategy, and is implemented by setting the uncertainty bonus to β=0 (Eq. 6). Effectively, this equates the value of options with their posterior mean $\text{MG}\left(x\right)=m\left(x|D_{t}\right)$.

The τ lesion model replaces the softmax choice function (Eq. 7) with an ϵ-greedy policy as an alternative mechanism for random exploration. Under this policy, with probability ϵ a random option is selected and with probability 1−ϵ, the option with the highest UCB value is chosen

$$p\left(x\right)=\left\{\begin{array}{cc} \text{arg}\;\text{max}\;\text{UCB}\left(x\right), & \text{with probability}\;1-\epsilon \\ 1/64, & \text{with probability}\;\epsilon \end{array}\right.$$ (12)

with the parameter ϵ estimated individually for each participant.

### Model cross-validation

Models’ predictive accuracy was assessed using leave-one-round-out cross-validation based on maximum likelihood estimation (*68*), with parameter bounds set to the range [exp(−5),exp(4)]. Specifically, we iteratively held out one round from the task, fitted each model to the remaining seven rounds, and then tested its ability to predict participants’ choices on the 25 trials of the holdout round. Predictive accuracy was quantified as the sum of negative log-likelihoods across all out-of-sample predictions. Individual parameter estimates for participants are based on averaging over the cross-validated maximum likelihood estimates.

The negative log-likelihoods served as the model evidence for the hierarchical Bayesian model selection based on protected exceedance probabilities (*42*) (Fig. 3A), and for quantifying predictive accuracy using a pseudo-*R*^2^ measure, where the summed log loss of each model is compared to a random baseline model. Accordingly, $R^{2}=0$ corresponds to chance performance and $R^{2}=1$ corresponds to theoretically perfect predictions

$$R^{2}=1-\frac{\text{log }L\left(M_{k}\right)}{\text{log }L\left(M_{\text{rand}}\right)}$$ (13)

For participant classification (Fig. 3B) we compared the models within each participant and chose the model with the highest *R*^2^ (or, equivalently, lowest log loss). In a supplementary analysis, we performed a model comparison on the group level likewise using the *R*^2^ measure. Consistent with both hierarchical Bayesian model selection and participant classification, the GP-UCB model achieved the highest *R*^2^ in each group (table S7 and fig. S6).

Note that because this measure is based on the likelihood of the exact chosen tile, it is inherently higher when behavior is concentrated on a small set of options (e.g., repeated exploitation of previously rewarding tiles, as controls and PD+ patients often did) than when choices are distributed across many plausible unrevealed options (e.g., broad exploration of novel tiles, as patients in the PD− group often did). Accordingly, the more exploratory behavior observed in the PD− group can yield lower absolute values of *R*^2^ even when the model captures the dominant strategy in each group. Therefore, relative model comparisons within each group, especially protected exceedance probabilities and comparisons against lesioned variants, are more informative than cross-group differences in absolute *R*^2^ values.

### Model simulations

To evaluate the performance of the GP-UCB model under different parameter settings we conducted computer simulations (Fig. 3D). Specifically, we fixed the amount of generalization to λ=1, corresponding to the true smoothness of the reward function in the used environments, and then varied the size of the uncertainty bonus (β) and the amount of random exploration (τ) over logarithmically spaced grids. Both parameters were sampled at 200 values between exp(−5) (≈0.0067) and exp(4) (≈54.6), resulting in a total of 40,000 unique (τ, β) combinations. For each parameter combination we simulated 1000 learners searching for rewards using the GP-UCB model, where environments were sampled (with replacement) from the set of 40 environments used in the behavioral study. In addition, we ran simulations with λ=0.5, which is closer to the mean group estimates; these simulations yielded comparable results (fig. S7).
