# Supplementary material for: Dopamine depletion in Parkinson’s increases directed but not random exploration
Source: Sci Adv. 2026 Aug 5;12(32):eaee6556. doi: 10.1126/sciadv.aee6556 (PMC13440411; doi:10.1126/sciadv.aee6556)
Supplement: Supplementary file 1 [file sciadv.aee6556_sm.pdf]

Science Advances
AAAS

Supplementary Materials for

Dopamine depletion in Parkinson's increases directed but not random exploration

Björn Meder *et al.*

Corresponding author: Björn Meder, bjoern.meder@hmu-potsdam.de

Sci. Adv. **12**, eaee6556 (2026)
DOI: 10.1126/sciadv.aee6556

This PDF file includes:

Supplementary Text
Figs. S1 to S7
Tables S1 to S7

These Supplementary Materials accompany the article "Dopamine depletion in Parkinson's increases directed but not random exploration" and contain the following elements:

- Supplementary Text
- Figs. S1 to S7
- Tables S1 to S7

Supplementary behavioral results

No performance differences in relation to round number or clinical indicators

We conducted hierarchical Bayesian regression analyses to examine whether performance differed across task rounds or as a function of clinical indicators.

First, we analyzed participants' mean reward per round as function of round number and group using a hierarchical linear regression. Group, round, and their interaction were specified as population-level ("fixed") effects, with group-level (random) intercepts for participants. This analysis showed only a difference between groups, but no performance differences across rounds (Table S1 and Figure S1).

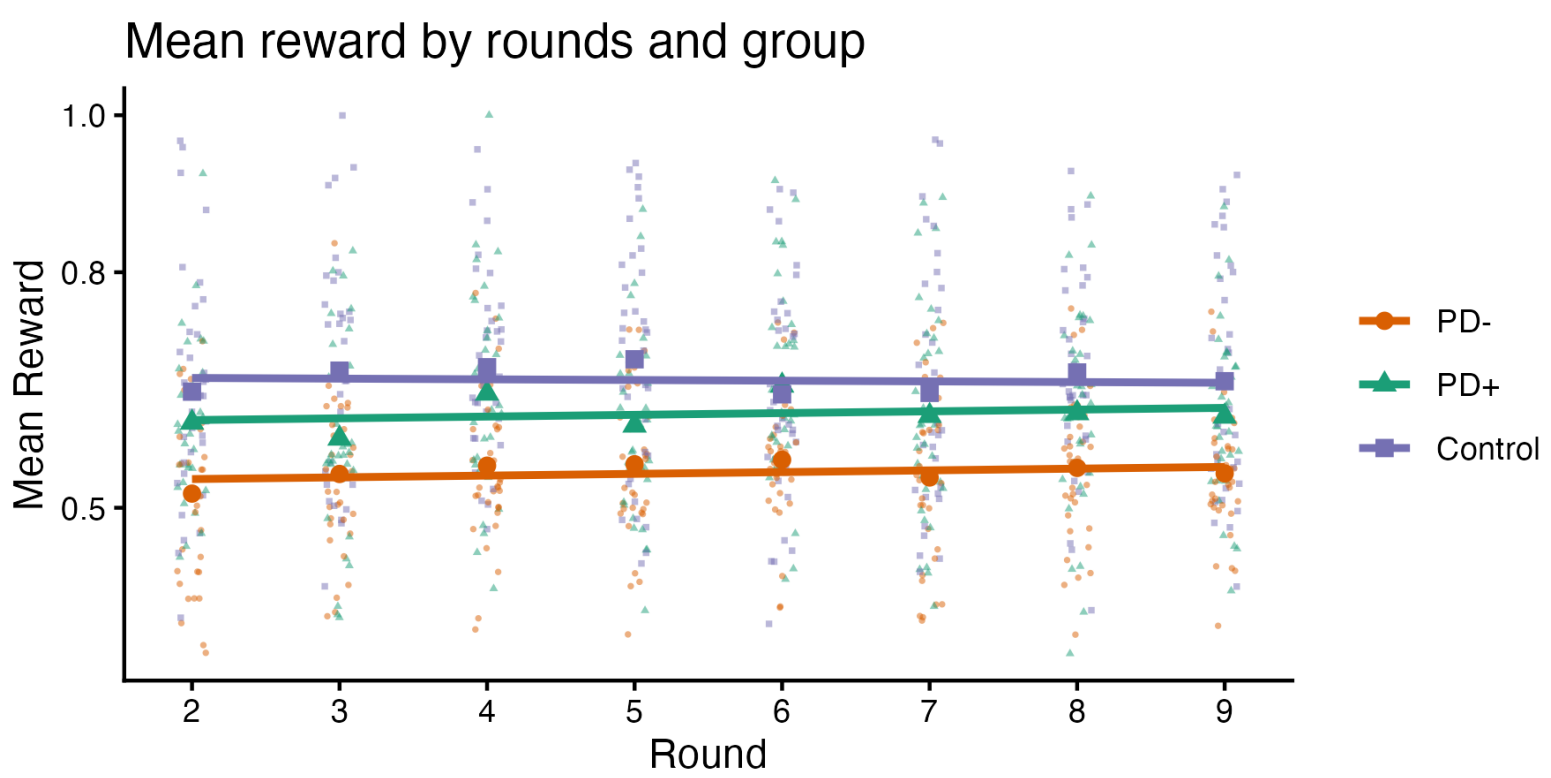

Fig. S1 Reward as a function of rounds. Each line is the population-level effect of a hierarchical Bayesian regression. Large markers show the group means in each round, small dots show individual participants.

Second, we ran a hierarchical Bayesian regression with reward per trial as dependent variable and group, BDI score, and MMSE score as population-level ("fixed") effects, and random intercepts for participants. This analysis also yielded only a substantial population-level effect of group, whereas the influence of both BDI and MMSE was minimal, with posterior estimates close to zero and the 95% HDI including zero (Table S2).

Finally, we ran a hierarchical Bayesian regression for PD patients only, with reward per trial as dependent variable and group, BDI, MMSE, and Hoehn-Yahr score as population-level predictors; and random intercepts for participants. Again, the analysis only yielded an influence of group, i.e. being tested on or off medication (Table S2). The coefficients for all clinical measures were very small, with all posterior means < 0.01 and their HDIs including zero.

In sum, in none of the analyses round number or clinical variables were related to performance. We therefore averaged rewards across rounds and did not further include the clinical indicators in the analyses.

Table S1 Bayesian linear multilevel regression: mean reward over rounds as a function of group.

	Estimate	95% HDI
**Population-level effects**		
Intercept	0.53	[0.49, 0.57]
Round	0.002	[−0.004, 0.008]
Group PD+	0.08	[0.02, 0.13]
Group Control	0.14	[0.08, 0.18]
Round × Group PD+	0.000004	[-0.008, 0.008]
Round × Group Control	-0.003	[-0.01, 0.01]
**Group-level effects**		
σ	0.11	[0.11, 0.12]
Observations	808	
N (participants)	101	

Note: Estimates are posterior means with 95% highest density intervals (HDI). Patients on medication (PD+) and controls are compared against PD− patients off medication. σ denotes the residual standard deviation.

Table S2 Bayesian linear multilevel regression: reward as a function of group, BDI, and MMSE.

	Estimate	95% HDI
**Population-level effects**		
Intercept	0.11	[-0.37, 0.57]
Group PD+	0.07	[0.04, 0.10]
Group Control	0.12	[0.09, 0.15]
BDI	0.0002	[-0.003, 0.004]
MMSE	0.02	[-0.001, 0.03]
**Group-level effects**		
σ	0.25	[0.24, 0.25]
Observations	20000	
N (participants)	100	

Note: Estimates are posterior means with 95% highest density intervals (HDI). Patients on medication (PD+) and controls are compared against PD− patients off medication. σ denotes the residual standard deviation. BDI and MMSE were entered as continuous predictors. One participant in the control group had a missing MMSE score and was therefore excluded from the analysis.

No medication-related difference in decision latency

To examine whether the behavioral differences between PD+ and PD− patients could reflect a general slowing of responding under dopamine depletion, we conducted exploratory analyses of response time, defined as the latency between consecutive clicks on the grid. Figure S2 shows subjects' median response time across all trials and rounds by group.

PD patients on and off medication did not differ in their median response time, $t(66) = -0.6$, $p = .569$, $d = 0.1$, $BF = .29$. Patients on medication did not differ from controls, $t(67) = -1.5$, $p = .149$, $d = 0.4$, $BF = .61$. Controls had slightly lower response times than PD− patients off medication, $t(67) = -1.9$, $p = .065$, $d = 0.5$, $BF = 1.1$.

To examine decision latency at the trial level, we fit a Bayesian hierarchical regression model predicting response time from group and distance to the previously selected tile, with a random intercept for participant (Table S4). Because response times were strictly positive and right-skewed, we used a lognormal link function. Controls responded faster than PD− patients, whereas the difference between PD patients on and off medication was small and the HDI included zero. As

Table S3 Bayesian linear multilevel regression including only patients with PD: reward as a function of group, BDI, MMSE, and Hoehn–Yahr score.

	Estimate	95% HDI
**Population-level effects**		
Intercept	0.31	[-0.22, 0.85]
Group PD+	0.07	[0.05, 0.10]
BDI	0.002	[-0.001, 0.006]
MMSE	0.007	[-0.01, 0.03]
Hoehn–Yahr	0.006	[-0.01, 0.03]
**Group-level effects**		
σ	0.24	[0.24, 0.25]
Observations	12200	
N (participants)	66	

Note: Estimates are posterior means with 95% highest density intervals (HDI). Patients on medication (PD+) are compared against PD− patients off medication. σ denotes the residual standard deviation. BDI, MMSE, and Hoehn–Yahr scores were entered as continuous predictors.

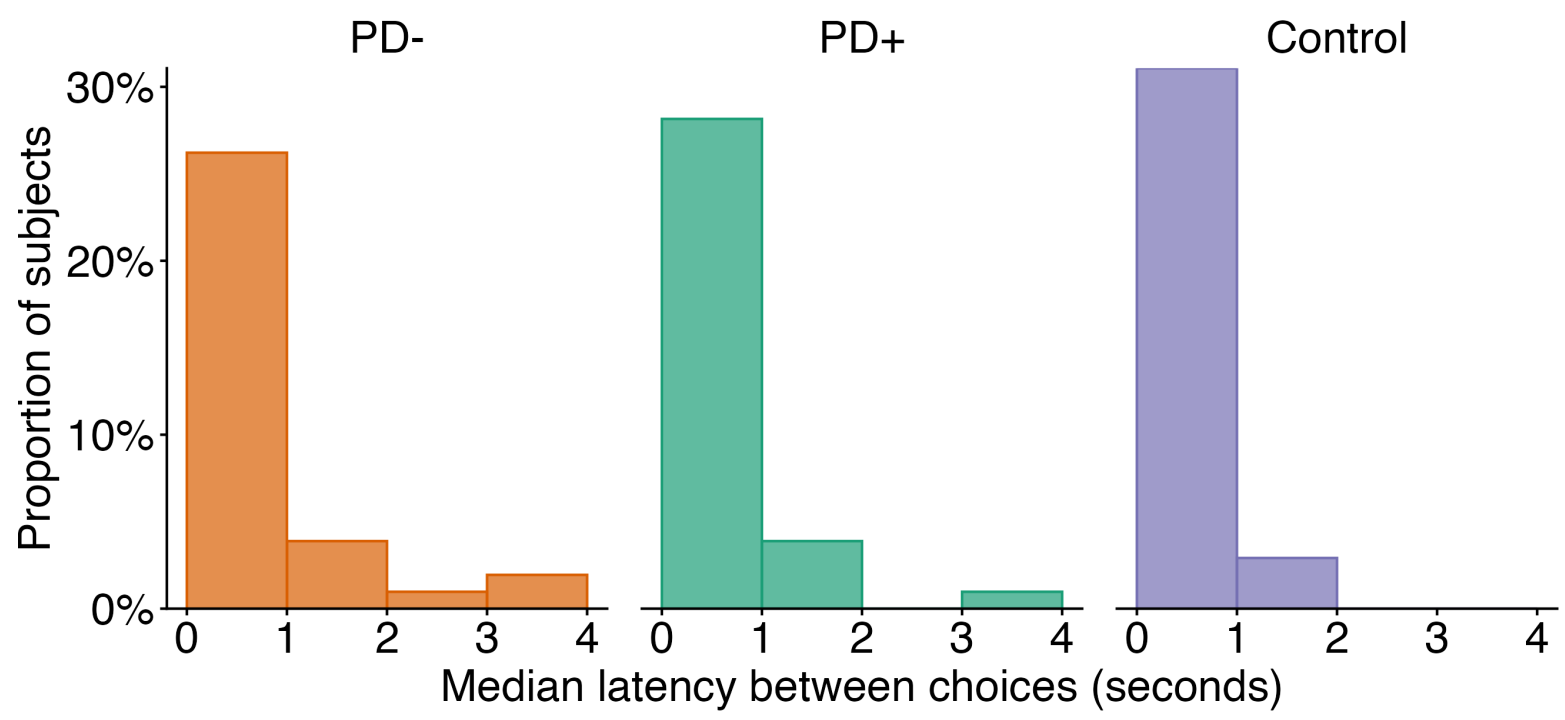

Fig. S2 Median response time between consecutive clicks across all trials and rounds.

expected, longer distances between consecutive responses were associated with longer latencies. The effect of distance on response time was slightly stronger in controls than in PD− patients, but the effect was small.

Overall, these analyses suggest that medication status was not associated with a difference in response latency within the PD sample, and that latency variation was driven more strongly by search distance than by group.

Bonus round: Reward judgments, confidence, and choices

The 10th and last round of the task as a “bonus round” in which participants first made 15 search decisions as in the previous rounds and then judged the expected rewards for 5 randomly chosen, previously unobserved tiles. For each tile, they also indicated how confident they were in their prediction (0-10). Subsequently, they chose one of the five tiles and continued the round as usual until the search horizon was exhausted.

Reward judgment error

Judgment error was measured as the absolute difference between a participant’s predictions and the mean of the true underlying Gaussian distribution. For each participant, we computed the mean absolute error by averaging over the five predictions. All three groups had lower judgment error than a random baseline (Figure S3; all $p < .001$). Controls had lower judgment error than PD− patients off medication ($t(67) = -2.7$, $p = .009$, $d = 0.6$, $BF = 5.0$). PD+ patients where

Table S4 Bayesian lognormal multilevel regression: decision latency as a function of search distance and group.

	Estimate	95% HDI
**Population-level effects**		
Intercept	6.55	[6.41, 6.69]
Distance	0.12	[0.11, 0.12]
Group PD+	-0.12	[-0.32, 0.08]
Group Control	-0.30	[-0.49, -0.11]
Distance × Group PD+	0.01	[-0.003, 0.01]
Distance × Group Control	0.03	[0.03, 0.04]
**Group-level effects**		
sd(Intercept)	0.41	[0.36, 0.47]
σ	0.47	[0.47, 0.48]
Observations	20600	
N (participants)	103	

Note: Estimates are posterior means with 95% highest density intervals (HDI). The outcome variable was decision latency in milliseconds. Because decision latencies were strictly positive and right-skewed, the model used a lognormal likelihood, thus regression coefficients are on the log scale of latency. Patients on medication (PD+) and controls are compared against PD− patients off medication. Distance refers to the Manhattan distance between consecutive choices.

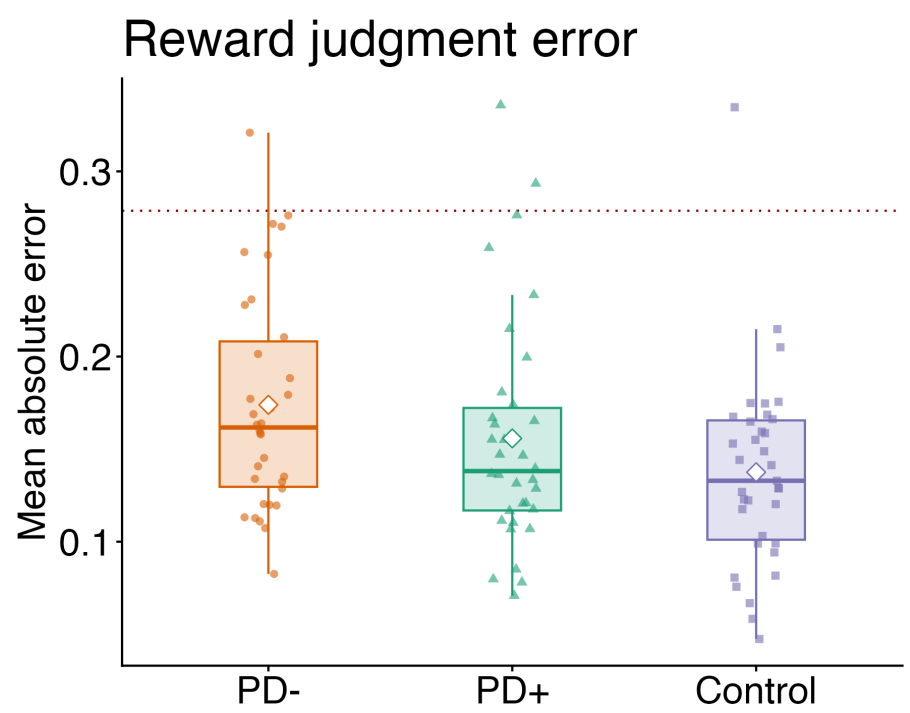

Fig. S3 Judgment error between participants' estimates and the true underlying expected rewards in the bonus round. For reference, the dashed line represents the expected error for a randomly choosing learner.

slightly worse than controls and slightly better than PD− patients, but did not differ from either group (both $p >$ .19).

To address whether the variability in prediction error in the bonus round was related to disease severity, we ran a Bayesian linear regression in the PD sample predicting mean absolute error from medication group, BDI, MMSE, and Hoehn–Yahr score (Table S5). Posterior estimates for all predictors were small and their 95% HDIs included zero, including for Hoehn–Yahr score. Thus, we found no evidence that variability in judgment error was systematically related to disease severity or other clinical indicators.

Judgment error and confidence

Across all participants and judgments, there was no relation between participants' judgment error and confidence in their judgments ($r = -.05$). A Bayesian hierarchical regression (Table S6) with confidence and group as population-level ("fixed") effects, and subject-wise random intercept showed only weak and inconsistent relationships across groups (Figure S4). The control group showed a weak negative relation (i.e., higher confidence was associated with lower judgment error),

Table S5 Bayesian regression: mean judgment error in the bonus round as a function of medication group and clinical indicators in the PD sample.

	**Estimate**	**95% HDI**
**Population-level effects**		
Intercept	-0.058	[-0.675, 0.555]
Group PD+	-0.021	[-0.052, 0.009]
Hoehn–Yahr score	0.001	[-0.023, 0.025]
BDI	-0.002	[-0.006, 0.002]
MMSE	0.009	[-0.011, 0.030]
**Distributional parameter**		
σ	0.062	[0.051, 0.073]
Observations	68	

Note: Estimates are posterior means with 95% highest density intervals (HDI). σ denotes the residual standard deviation.

the PD+ group a weak positive trend (i.e., lower confidence was associated with higher judgment error), and the PD− group showed no association.

Table S6 Bayesian multilevel regression: judgment error as a function of group, confidence, and their interaction.

	**Estimate**	**95% HDI**
**Population-level effects**		
Intercept	0.180	[0.151, 0.209]
Confidence	-0.002	[-0.008, 0.004]
Group PD+	-0.039	[-0.085, 0.008]
Group Control	-0.001	[-0.052, 0.048]
Confidence × Group PD+	0.005	[-0.004, 0.014]
Confidence × Group Control	-0.007	[-0.018, 0.002]
**Group-level effects**		
σ	0.115	[0.107, 0.122]
Observations	515	
N (participants)	103	

Note: Estimates are posterior means with 95% highest density intervals (HDI). σ denotes the residual standard deviation. The model includes interactions between group and confidence (howSecure).

Judged rewards of chosen and unchosen tiles

After making reward judgment for the five tiles, participants could choose one of them and then continued the round as usual. (Figure S5) compares the judged rewards for chosen and unchosen tiles, where the predicted reward for unchosen tiles is based on averaging across all four tiles that were not chosen. In the control group, judged rewards for chosen tiles were higher than for unchosen tiles ($t(34) = 2.9$, $p = .007$, $d = 0.6$, $BF = 6.1$), as well as in PD+ patients ($t(33) = 2.1$, $p = .047$, $d = 0.3$, $BF = 1.2$). No difference was obtained for PD− patients off medication ($p > .8$). In none of the groups average confidence differed between chosen and unchosen tiles (all $p > .3$).

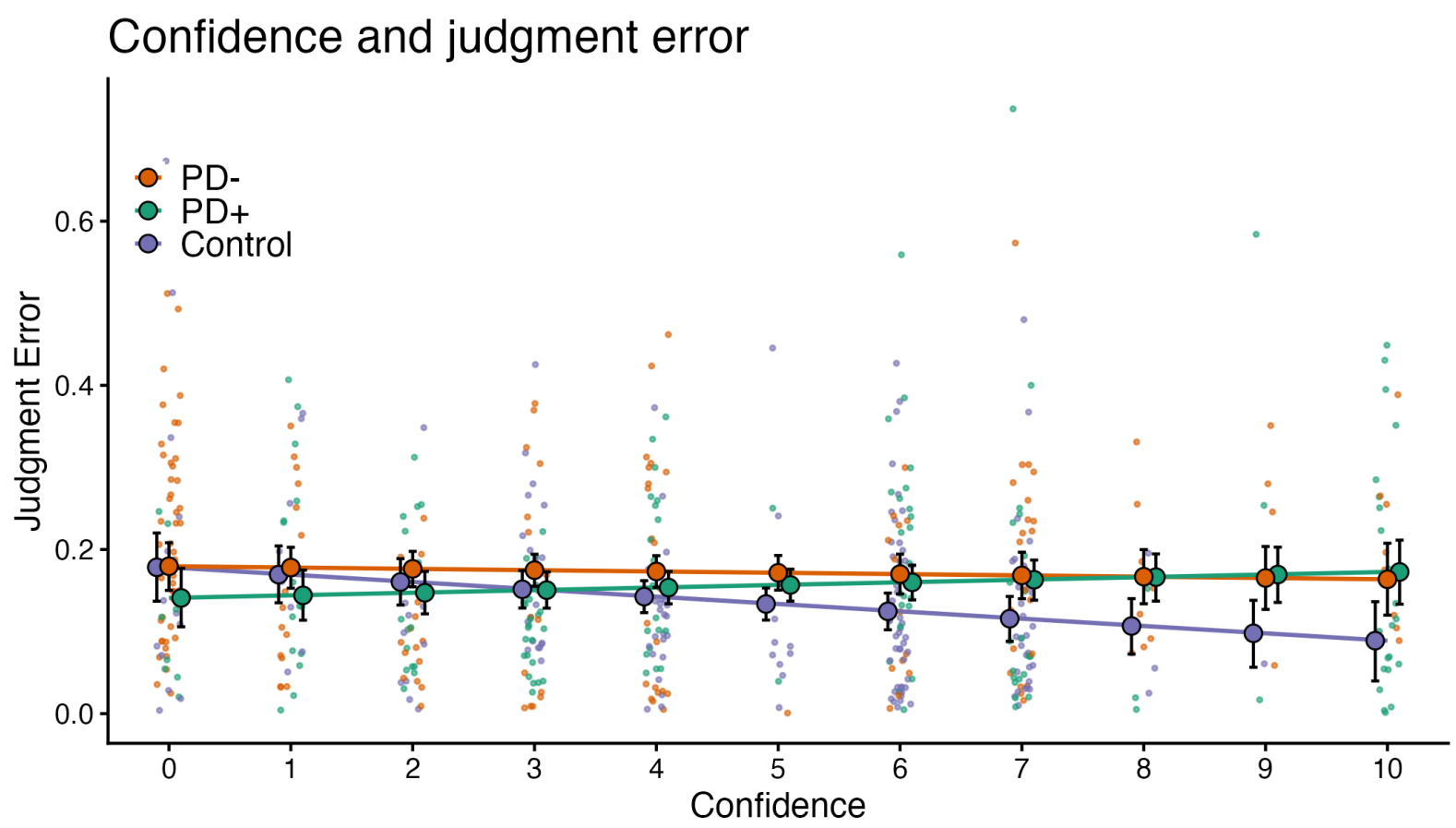

Fig. S4 Predictions of a Bayesian hierarchical regression with reward judgment error as a function of confidence and group. Large dots show posterior means, with error bars denoting 95% credible intervals. Small dots show observed individual data points.

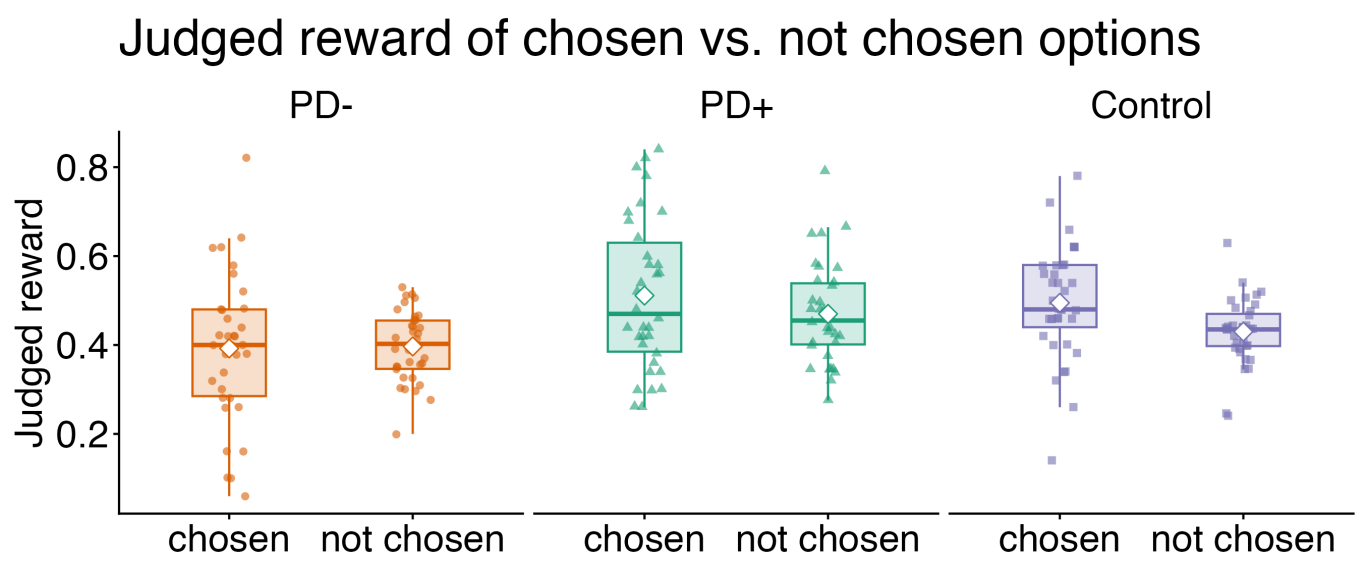

Fig. S5 Participants' reward judgments for chosen versus not chosen options in the bonus round. Small dots show individual data points.

Supplementary computational modeling results

Model comparison: All components of the GP-UCB model are critical to explain behavior

The predictive accuracy of the computational models was evaluated through leave-one-round-out cross-validation using maximum likelihood estimation (*69*). Predictive accuracy was quantified as the sum of negative log-likelihoods across all out-of-sample predictions.

In addition to the hierarchical Bayesian model selection (Fig. 3A) and individual participant classification (Fig. 3B), we also compared the predictive accuracy R^2 across models and groups. In each group, the GP-UCB model achieved the highest predictive accuracy compared to the three lesioned models (Fig. S6). Table S7 shows the results of t-tests comparing the mean R^2 of the GP-UCB model to each lesioned model, showing that the GP-UCB model consistently outperformed all lesioned variants across groups.

Simulated performance of the GP-UCB model

To evaluate how well different parameter settings balance exploration and exploitation, we conducted simulations with the GP-UCB model. Figure 3D in the main text shows participant parameters in relation to the performance landscape with the length-scale parameter of the RBF kernel (Eq. 2) set to $\lambda = 1$, the true amount of spatial correlation in the used environments.

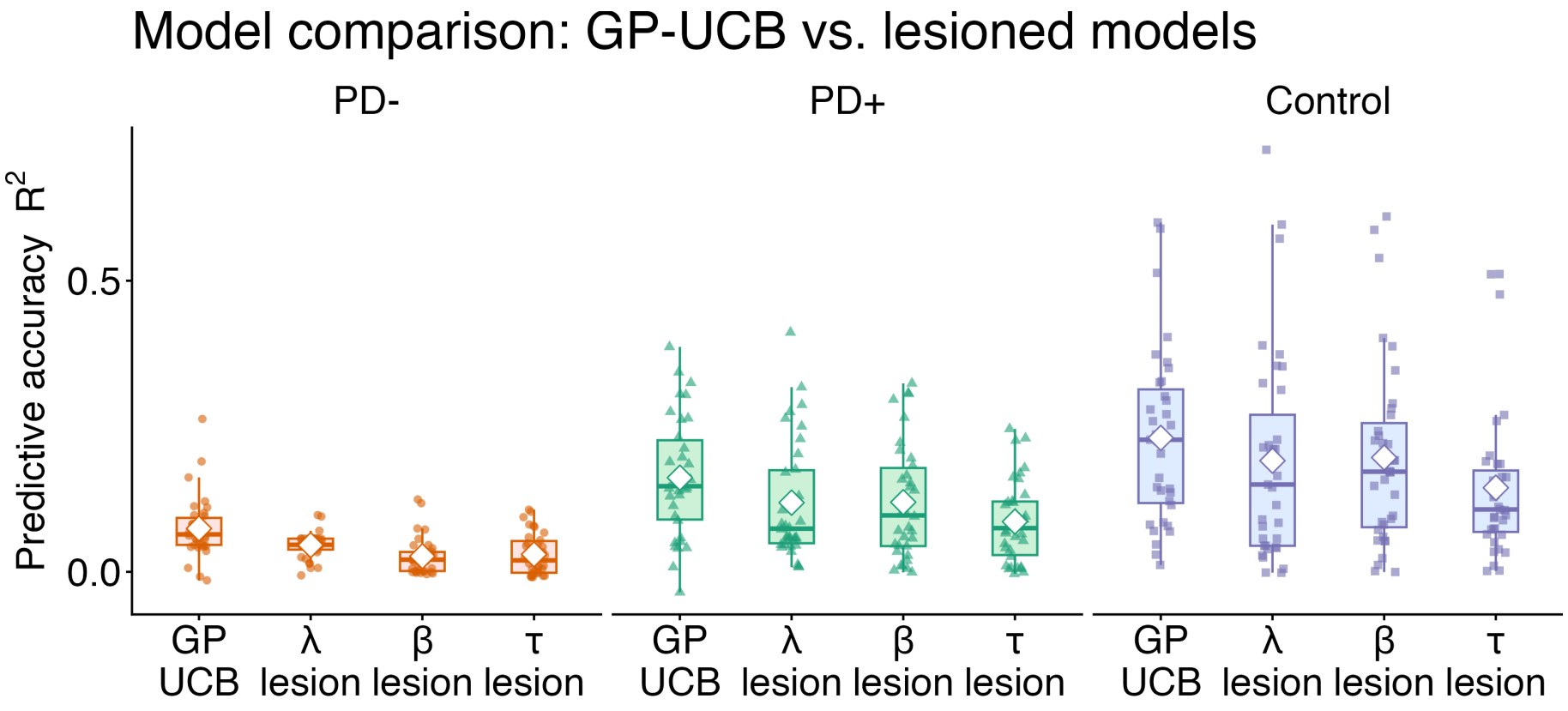

Fig. S6 Predictive model accuracy. Small dots show individual data points.

Group	Comparison	n	t	p	d	BF
PD−	λ lesion	34	3.8	$p < .001$	0.7	$BF = 47$
PD−	β lesion	34	5.8	$p < .001$	1.1	$BF > 100$
PD−	τ lesion	34	5.2	$p < .001$	1.0	$BF > 100$
PD+	λ lesion	34	3.4	$p = .002$	0.4	$BF = 18$
PD+	β lesion	34	3.8	$p < .001$	0.4	$BF = 58$
PD+	τ lesion	34	8.7	$p < .001$	0.9	$BF > 100$
Control	λ lesion	35	3.2	$p = .003$	0.2	$BF = 12$
Control	β lesion	35	3.5	$p = .001$	0.2	$BF = 28$
Control	τ lesion	35	7.9	$p < .001$	0.6	$BF > 100$

Table S7 Pairwise comparisons of R^2 values between GP-UCB model and lesioned variants across groups.

We additionally conducted simulations with $\lambda = 0.5$, which is closer to the mean group estimates of λ, $M_{\text{PD}-} = 0.53$, $M_{\text{PD}+} = 0.56$, $M_{\text{Control}} = 0.64$. Again, we systematically varied the amount of random exploration (τ) and the size of the uncertainty bonus (β) over logarithmically spaced grids. Both parameters were sampled at 200 values between $\exp(-5)$ (≈ 0.0067) and $\exp(4)$ (≈ 54.6), resulting in a total of 40,000 unique (τ, β) combinations. For each parameter combination we simulated 1000 learners searching for rewards using the GP-UCB model, where environments were sampled (with replacement) from the set of 40 environments used in the behavioral study.

Figure S7 depicts the resulting performance landscape together with participants' parameter estimates for β and τ. The results are similar to performance landscape for $\lambda = 1$: Parameter values of controls and PD+ patients are closer to the optimal region compared to those of PD− patients off medication. For the latter, especially the too high β valued shift parameter estimates into low-reward regions of the landscape.

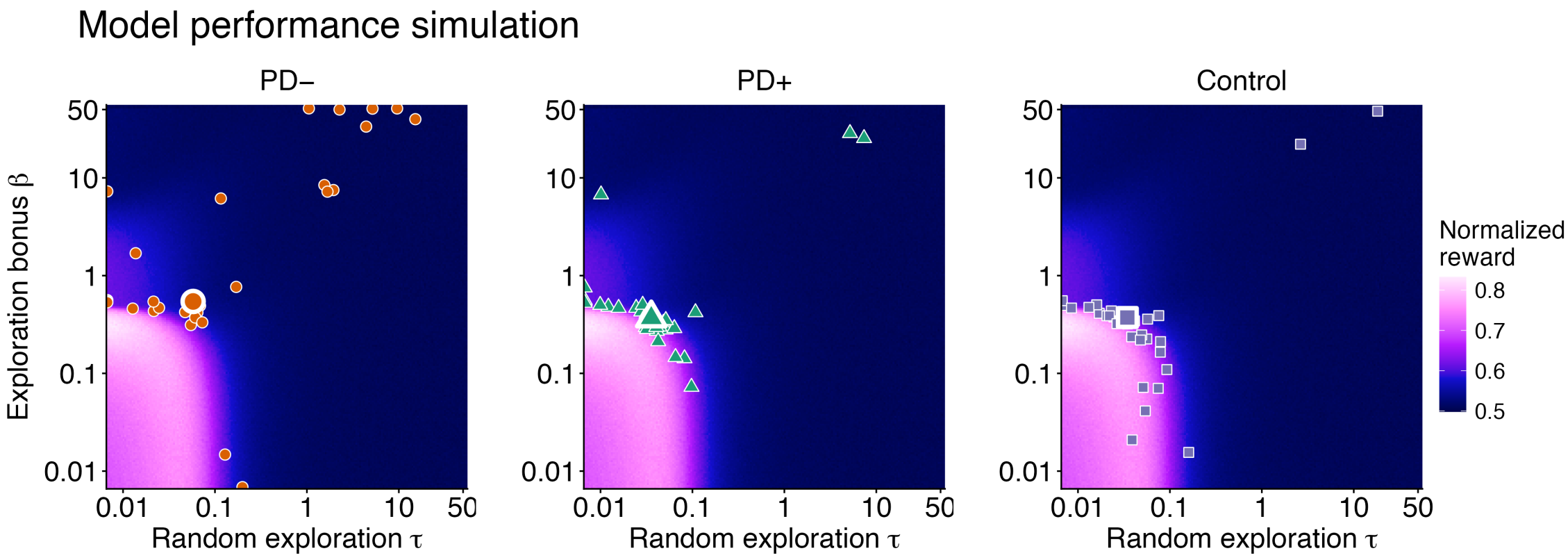

Fig. S7 Performance landscape of the GP-UCB model across different amounts of random exploration τ and uncertainty-directed exploration β. The amount of generalization was fixed to λ=0.5 and β and τ were varied, with 1000 simulated learners per parameter combination. Each dot shows one participant; the larger markers indicate the group median.
